# The role of virulence factors in the outcome of staphylococcal peritonitis in CAPD patients

**DOI:** 10.1186/1471-2334-9-212

**Published:** 2009-12-22

**Authors:** Pasqual Barretti, Augusto C Montelli, Jackson EN Batalha, Jacqueline CT Caramori, Maria de Lourdes RS Cunha

**Affiliations:** 1 Department of Internal Medicine, Botucatu School of Medicine, UNESP, Botucatu, Sao Paulo, Brazil; 2 Department of Microbiology and Immunology, Biosciences Institute, UNESP, Botucatu, Sao Paulo, Brazil

## Abstract

**Background:**

Peritonitis continues to be the most frequent cause of peritoneal dialysis (PD) failure, with an important impact on patient mortality. Gram-positive cocci such as *Staphylococcus epidermidis*, other coagulase-negative staphylococci (CoNS), and *Staphylococcus aureus *are the most frequent etiological agents of PD-associated peritonitis worldwide. The objective of the present study was to compare peritonitis caused by *S. aureus *and CoNS and to evaluate the factors influencing outcome.

**Methods:**

Records of 86 new episodes of staphylococcal peritonitis that occurred between 1996 and 2000 in the Dialysis unit of a single university hospital were studied (35 due to *S. aureus*, 24 to *S. epidermidis *and 27 to other CoNS). The production of slime, lipase, lecithinase, nuclease (DNAse), thermonuclease (TNAse), α- and β-hemolysin, enterotoxins (SEA, SEB, SEC, SED) and toxic shock syndrome toxin-1 (TSST-1) was studied in *S. aureus *and CoNS. Antimicrobial susceptibility was evaluated based on the minimal inhibitory concentration determined by the E-test. Outcome predictors were evaluated by two logistic regression models.

**Results:**

The oxacillin susceptibility rate was 85.7% for *S. aureus*, 41.6% for *S. epidermidis*, and 51.8% for other CoNS (p = 0.001). Production of toxins and enzymes, except for enterotoxin A and α-hemolysin, was associated with *S. aureus *episodes (p < 0.001), whereas slime production was positive in 23.5% of CoNS and 8.6% of *S. aureus *strains (p = 0.0047). The first model did not include enzymes and toxins due to their association with *S. aureus*. The odds of resolution were 9.5 times higher for *S. epidermidis *than for *S. aureus *(p = 0.02) episodes, and were similar for *S. epidermidis *and other CoNS (p = 0.8). The resolution odds were 68 times higher for non-slime producers (p = 0.001) and were not influenced by oxacillin resistance among vancomycin-treated cases (p = 0.89). In the second model, the resolution rate was similar for *S. aureus *and *S. epidermidis *(p = 0.70), and slime (p = 0.001) and α-hemolysin (p = 0.04) production were independent predictors of non-resolution.

**Conclusion:**

Bacterial species and virulence factors rather than antibiotic resistance influence the outcome of staphylococcal peritonitis.

## Background

Peritonitis continues to be the most frequent cause of peritoneal dialysis (PD) failure [1], and has an important impact on patient mortality [2]. Gram-positive cocci such as *Staphylococcus epidermidis*, other coagulase-negative staphylococci (CoNS), and *Staphylococcus aureus *are the most frequent etiological agents of PD-associated peritonitis worldwide [3].

Previous studies comparing the outcome of peritonitis caused by *S. aureus *and CoNS have shown a lower resolution rate and a higher frequency of complications in the former [4-8]. Perez-Fontan et al [2] observed a mortality rate of 15.2% for *S. aureus *episodes and of only 0.5% for CoNS episodes. These findings are in contrast to the susceptibility profile observed for CoNS. According to Kim et al [9], the frequency of methicillin-resistance among CoNS increased from 18.4% in 1992-1993 to 41.7% in 2000-2001. Similar results have been reported by other investigators [10]. Previous data from our group showed that oxacillin resistance does not influence the outcome of staphylococcal peritonitis [8].

In addition to species and antibiotic resistance, other factors related to the causal agent may influence the prognosis of peritonitis. Recurrence of CoNS infections is frequently observed and has been suggested to be associated with the presence of a biofilm in the peritoneal catheter [11]. Biofilm formation is related to the production of an extracellular mucoid polysaccharide, called slime, which permits microorganisms to adhere to plastic surfaces [12]. Kristinsson et al [13] reported a higher recurrence rate of peritonitis for slime-positive strains compared to slime-negative ones, whereas Alexander and Rimland [14] did not observe a relationship between slime production and peritonitis outcome. In our unit, slime production was found to be an independent risk factor for the non-resolution of CoNS peritonitis [15].

The production of enzymes and toxins is a well-known fact in *Staphylococcus *species, particularly *S. aureus*. Proteases, lipases, nucleases, and collagenases convert tissue components into nutrients, facilitating bacterial growth and invasion [16], while toxic shock syndrome toxin (TSST-1) and enterotoxins have effects such as superantigenicity, pyrogenicity, toxicity and direct damage to endothelial [6].

Although these products are potential virulence factors in staphylococcal PD-associated peritonitis, their influence on the clinical outcome of these infections is unknown. The objective of the present study was to compare the capacity of traditional clinical and bacteriologic and selected virulence factors such as production of slime, enzymes and toxins to predict the outcome of new peritonitis episodes caused by *S. aureus*, *S. epidermidis *and other CoNS.

## Methods

### Data collection and definitions

The present study was approved by the institutional Ethics Committee. All episodes of continuous ambulatory peritoneal dialysis (CAPD)-associated peritonitis caused by staphylococcal species between January 1996 and December 2000 were reviewed. This period was chosen because a single antibiotic protocol based on the 1996 Update of the International Society for Peritoneal Dialysis [17] was used. After this period, new guidelines were proposed [18] and adopted in our unit. The diagnosis of peritonitis was made when at least two of the following criteria were present: (a) presence of a cloudy peritoneal effluent, (b) abdominal pain, (c) dialysate white cell count higher than 100/μL, with at least 50% polymorphonuclear cells, and (d) positive culture of peritoneal effluent [17].

Only cases considered to be new episodes, i.e., a patient's first peritonitis or an episode diagnosed at least 28 days after completion of the last peritonitis treatment, were included in the study [17]. Thus, 86 of 122 diagnosed staphylococcal peritonitis episodes were analyzed. Exclusion criteria were staphylococcal peritonitis within 28 days prior to presentation, presence of concomitant exit site or tunnel infections, incomplete clinical data, concomitant antibiotic use for other indications, and use of an empirical antibiotic protocol other than the combination of cefazolin and amikacin.

Resolution was defined as the disappearance of signs and symptoms within 96 h after the beginning of antibiotic therapy and a negative peritoneal fluid culture at least 28 days after treatment completion [7]. Relapse was defined as an episode with the same organism or a negative culture result that occurs within 28 days of completion of antibiotic therapy for a prior episode [17]. Non-resolution was the term used for cases presenting initial non-resolution, relapse, peritoneal catheter removal, or death.

The following information was recorded for each case: (1) episode: date, clinical findings, treatment, outcome (resolution, relapse, catheter removal, or death); (2) presence of diabetes mellitus; (3) demographic data: age, gender, and race (Caucasian, non-Caucasian), dialysis treatment time; (4) exchange system (standard or double bag).

### Clinical management

All episodes were treated according to the local protocol adapted from the third report of the Ad Hoc Advisory Committee on Peritonitis Management for staphylococcal episodes [17]. Patients were treated within 24 h of the onset of the first clinical signs or symptoms and antibiotic therapy was started with 500 or 750 mg/L cefazolin, ip (patients with urine volume >500 mL/24 h) and 250 mg/L amikacin, ip, as loading dose, followed by 500 or 750 mg/L cefazolin and 2 mg/kg amikacin per day in the last PD bag. Therapy was evaluated as soon as the culture results were available. For oxacillin-susceptible cocci, cefazolin was maintained, whereas for oxacillin-resistant cocci cefazolin was replaced with 1 g/L vancomycin, ip, administered at intervals of 5 (patients with urine volume >500 mL/24 h) or 7 days. Amikacin was discontinued in both cases. Vancomycin was also administered to patients who presented no clinical improvement within the first 96 h of antibiotic treatment, although the microbiological tests revealed oxacillin susceptibility. The duration of antibiotic therapy was 14 days for CoNS episodes and 21 days for *S. aureus *episodes.

### Microbiological tests

Microbiological samples were stored in a culture collection. The isolates obtained from clinical specimens were seeded onto blood agar and gram-stained to confirm purity and to determine morphology and specific color. The isolates were then submitted to catalase and coagulase tests. CoNS were identified using the simplified biochemical test scheme proposed by Kloos and Schleifer [19] and Kloos and Bannerman [20].

*In vitro *susceptibility was evaluated based on the minimal inhibitory concentration determined by the E-test (AB Biodisk, Solna, Sweden), a quantitative method that uses a transparent strip of inert plastic containing drug concentrations ranging from 0.002 to 256 μg/mL. The proportion of strains susceptible to each drug was defined based on the 2005 CLSI breakpoints [21]. Strains presenting intermediate values were considered to be resistant.

### Determination of the production of pathogenic factors

#### Slime

Slime production was evaluated according to Christensen et al [22]. Colonies of CoNS isolated on blood agar were inoculated into tubes measuring 12.0 × 75.0 mm and containing 2.0 mL trypticase soy broth (TSB) and incubated for 48 h at 37°C. Next, 1.0 mL 0.4% trypan blue or toluidine blue O solution was added to the tubes. After gentle shaking to guarantee staining of the material adhered to the inner surface of the tubes, the dye was discarded. A positive result was defined as the presence of a layer of stained material adhered to the inner wall of the tubes. The presence of a colored ring only at the liquid-air surface was classified as negative.

#### Alpha- and beta-hemolysin

Production of α- and β-hemolysin or cytolytic toxins was determined on plates containing blood agar base supplemented with 5% rabbit blood and 5% sheep blood, respectively. The plates were incubated for 24 h at 37°C. The formation of hemolysis zones around the isolated colonies indicated a positive result.

#### Lipase and lecithinase

Lipolytic activity was determined on plates containing blood agar base enriched with 0.01% CaCl_2_:2H_2_O and 1% Tween 80. A positive result was defined as the formation of opacity around the colony after incubation for 18 h at 37°C, followed by incubation at room temperature for 24 h [23]. The production of lecithinase was evaluated using Baird-Parker medium. The formation of an opaque halo around the colony indicated a positive result [24].

#### DNAse and TNAse

Nuclease (DNAse) and thermonuclease (TNAse) were determined by the metachromatic toluidine blue O agar diffusion-DNA technique according to Lachica et al [25]. For the detection of DNAse, supernatants were obtained from CoNS cultures in BHI broth previously incubated for 24 h at 37°C and centrifuged at 8000 *g *for 10 min at 4°C. The culture supernatant was first heated in a water bath for 20 min and then placed in the wells for the detection of TNAse. Nuclease (DNAse and TNAse) activity was determined by measuring the diameter of pink halos (mm) formed on the medium. Positive results were interpreted by comparison of the halos with those obtained for a standard DNAse- and TNAse-positive *S. aureus *strain (ATCC 25923). Culture supernatants obtained by the sac culture method of Donnelly et al [26], as described below, were also tested for DNAse and TNAse production.

#### Enterotoxins and toxic shock syndrome toxin-1

The sac culture method for toxin production [26] was used to determine the toxigenic profile of the strains. Dialysis sacs filled with 50 mL double-concentrated BHI broth were placed in U-shaped Erlenmeyer flasks and autoclaved for 15 min at 121°C. A loopful of organisms was added to 18 mL sterile 0.2 M phosphate buffer in 0.9% NaCl, pH 7.4. After incubation for 24 h at 37°C on a shaker at 200 rpm, the cultures were centrifuged at 8000 *g *for 10 min at 4°C and the supernatants obtained were stored at -20°C until the time of use. The extracellular products were detected by reverse passive latex agglutination (RPLA) according to Shingaki et al [27], using the SET-RPLA-T900 and TST-RPLA-TD940 kits (Oxoid Diagnostic Reagents) for the detection of enterotoxins A (SEA), B (SEB), C (SEC) and D (SED) and TSST-1, respectively. Culture supernatants were first treated with 5% (v/v) normal rabbit serum or 5% purified rabbit IgG to block nonspecific reactions. Samples that presented nonspecific reactions even after this treatment were filtered through a Millipore membrane (8.0 μm) and, if necessary, diluted 1:10 with 0.02 M phosphate buffer in 0.9% NaCl, pH 7.4. A positive reaction was classified as (+), (++) and (+++) according to the agglutination pattern described by the manufacturer of the kit. The formation of a rose button was interpreted as a negative result.

### Statistical analysis

Continuous variables were compared using the unpaired t-test or nonparametric Mann-Whitney U-test. Binary variables were compared by the chi-square or Fisher's exact test. Multivariate analysis by logistic regression was used to test for factors that independently predicted the outcome of a peritonitis episode, with outcome being classified into two mutually exhausted and exclusive results (resolution or non-resolution). A model was adopted that would incorporate the effect of all factors-interest and control - on the outcome of infection. All baseline demographic, clinical, and microbiological variables, including age, gender, diabetic status, dialysis duration, exchange system, use of vancomycin, *Staphylococcus *species, oxacillin susceptibility and pathogenic factor production, were included in the model. A p value less than 0.05 was considered to be significant.

## Results

Eighty-six new CAPD-associated staphylococcal peritonitis episodes occurred in 63 patients between 1996 and 2000. Forty-three of the patients were females, 39 were Caucasians, and 28 had diabetes. The distribution of patients according to age was as follows: birth to 20 years (n = 4), 21 to 40 years (n = 12), 41 to 59 years (n = 22), and 60 years or older (n = 22). Treatment time on CAPD was less than one year in 28 patients and longer than one year in 35. Forty-four patients used a double bag system and 19 used a standard bag exchange system.

### Microbiological investigation

Thirty-five episodes were caused by *S. aureus *and 51 by CoNS. Among CoNS, *S. epidermidis *was the most frequent species (24 cases), followed by *S. haemolyticus *(11 cases) and other species (16 cases) (Table 1).

**Table 1:** Causative agent of the 86 new episodes of peritonitis caused by *S. aureus *and coagulase-negative staphylococci.

Microorganism	No. of strains (%)
*S. aureus*	35
CoNS	51 (100)
*S. epidermidis*	24 (47)
*S. haemolyticus*	11 (21.5)
*S. warneri*	5 (9.8)
*S. hominis*	5 (9.8)
*S. xylosus*	2 (3.9)
*S. cohnii*	2 (3.9)
*S. simulans*	1 (1.9)
*S. lugdunensis*	1 (1.9)

CoNS: coagulase-negative staphylococci.

Oxacillin susceptibility was observed in 30 (85.7%) of the 35 episodes due to *S. aureus*, in 10 (41.7%) of the 24 episodes due to *S. epidermidis*, and in 12 (44.4%) of the 27 episodes due to other CoNS (*p *= 0.0002). Five cases of intermediate susceptibility were detected among CoNS and one among *S. aureus *strains.

Positive slime production was observed in three cases due to *S. aureus *(8.6%) and in nine (17.6%) due to CoNS (*p *= 0.345), including *S. epidermidis *in four, *S. haemolyticus *in two, *S. warneri *in two, and *S. lugdunensis *in one.

With respect to toxigenic profile, 25 (71.4%) of the 35 *S. aureus *strains were toxin producers, whereas only seven (13.7%) of the 51 CoNS strains produced some type of toxin (*p *< 0.00001). The rate of enzyme production was higher in *S. aureus *strains than in *S. epidermidis *or other CoNS, except for α-hemolysin whose production was similar in all strains. The rates of toxin and enzyme production by *S. aureus*, *S. epidermidis *and other CoNS species are shown in Table 2.

**Table 2:** Rates of toxin and enzyme production by *S. aureus, S. epidermidis *and other coagulase-negative staphylococci strains isolated from 86 new episodes of peritonitis.

	*S. aureus*	*S. epidermidis*	Other CoNS	p
**Toxins**				
SEA	4 (11.4)	1 (4.1)	- (-)	0.15
SEB	12 (34.3)	- (-)	2 (7.4)	0.0006
SEC	8 (22.8)	- (-)	6 (22.2)	0.04*
SED	- (-)	- (-)	- (-)	-
TSST-1	12 (34.3)	1 (4.1)	- (-)	0.0008
**Enzymes**				
α-Hemolysin	17 (48.6)	8 (33.3)	8 (29.8)	0.26
β-Hemolysin	29 (82.3)	6 (25)	7 (25.9)	< 0.00001
Lipase	34 (97.1)	4 (16.7)	5 (18.5)	< 0.00001
Lecithinase	34 (97.1)	2 (8.3)	6 (22.2)	< 0.00001
DNAse	34 (97.1)	- (-)	4 (14.8)	< 0.00001
TNAse	34 (97.1)	- (-)	4 (14.8)	< 0.00001

CoNS: coagulase-negative staphylococci; SEA, SEB, SEC, and SED: enterotoxins A, B, C and D, respectively; TSST-1: toxic shock syndrome toxin; * *S. aureus *vs. *S. epidermidis *and other CoNS vs. *S. epidermidis*.

### Clinical outcome

Overall, 57 (66.3%) episodes were resolved, 14 (16.3%) relapsed, 12 (13.9%) required removal of the catheter, and three (3.5%) progressed to death. Among the 35 *S. aureus *cases, 17 (48.6%) were resolved, eight (22.8%) relapsed, seven (20%) required catheter removal, and three (8.6%) progressed to death. Regarding CoNS episodes, 40 (78.4%) were resolved, six (11.8%) relapsed, and five (9.8%) required catheter removal. Among these 11 non-resolved episodes, seven were due to *S. epidermidis*, two to *S. haemolyticus*, and two to other CoNS. There were significantly more CoNS cases resolved than *S. aureus *episodes (p < 0.001).

There were 52 episodes involving oxacillin-susceptible strains, 32 (61.5%) of them were resolved, 10 (19.2%) relapsed, and 10 (19.2%) required catheter removal. Thirty-four infections were caused by oxacillin-resistant strains, 20 (58.8%) of them were resolved, nine (26.5%) relapsed, and five (14.7%) required catheter removal. The resolution rate was similar for oxacillin-susceptible and -resistant strains (p = 0.9713).

Vancomycin was used in 56 episodes, 18 caused by *S. aureus *and 38 by CoNS. This antibiotic was prescribed because of bacterial resistance in 28 cases, lack of improvement in 20, and other undefined causes in eight. The time between diagnosis and the first vancomycin dose was 3.7 ± 1.7 days for *S. aureus *episodes and 3.9 ± 1.2 days for CoNS episodes (p = 0.793).

Two regression models were constructed. In Model 1 slime production was the only pathogenic factor, whereas in Model 2 the production of toxins and enzymes was included. Only lecithinase, α-hemolysin and TNAse were included since associations were observed between lecithinase and lipase, α-hemolysin and β-hemolysin, and TNAse and DNAse (p = 0.001). All toxins were included, except for toxin D which was not produced by any of the strains. Since an interaction effect was observed between *S. aureus *species and oxacillin susceptibility (p = 0.01), the influence of oxacillin susceptibility was evaluated at each vancomycin treatment co-variable level in both models.

In Model 1 (Table 3), controlling for co-variables, the odds of resolution was not influenced by host factors such as age, gender, diabetes, exchange system, or CAPD treatment time. The odds of resolution were 9.5 times higher for *S. epidermidis *than for *S. aureus *episodes (*p *= 0.0263), whereas similar resolution odds were observed for *S. epidermidis *and the other CoNS (*p *= 0.085). Among strains isolated from infections treated with vancomycin, no significant difference was observed between oxacillin-susceptible and -resistant strains (p = 0.89). In contrast, among strains isolated from infections not treated with vancomycin, there was a significant difference between strains susceptible and resistant to oxacillin (p = 0.0113). In this case, the chance of cure of infections caused by oxacillin-susceptible strains was 137 times higher than that of infections caused by resistant strains. With respect to slime production, the chance of cure of infections caused by non-producers was estimated to be up to 68 times higher than that of infections caused by slime producers (p = 0.0015).

**Table 3:** Odds comparison of peritonitis resolution by logistic regression analysis (Model 1).

Factor	Log (Odds)	p	Odds ratio (95% CI)
Age (birth to 20 years/>60 years)	-0.0510	0.96	
Age (21 to 40 years/>60 years)	1.7097	0.09	
Age (41 to 59 years/>60 years)	0.7511	0.84	
Gender (male/female)	1.2523	0.19	
Race (Caucasian/non-Caucasian)	-0.0818	0.92	
Diabetes mellitus (no/yes)	-0.6709	0.12	
System (standard/double bag)	1.2585	0.22	
Treatment time on CAPD			
<1 year	0.2074	0.89	
>1 year	0.9547	0.21	
Treatment with vancomycin			
Oxacillin susceptible/oxacillin resistant	-0.1070	0.89	
No treatment with vancomycin			
Oxacillin susceptible/oxacillin resistant	1.9440	0.01	137 (3.0; 6,202.4)
Etiological agent			
*S. epidermidis/S. aureus*	2.2548	0.02	9.5 (1.3; 69.6)
Other CoNS/*S. epidermidis*	-1.6113	0.08	
Slime production (no/yes)	4.2139	0.001	68 (5.0; 914.8)

CoNS: coagulase-negative staphylococci; CI: 95% Confidence Interval for the true Odds Ratio.

As observed for the first model, in Model 2 (Table 4) resolution odds were not influenced by host factors. Among strains isolated from infections treated with vancomycin, no significant difference was observed between those susceptible and resistant to oxacillin (p = 0.1523), whereas there was a significant difference among strains not treated with vancomycin (p = 0.0039). With respect to slime production, the chance of cure of infections caused by non-producers was estimated to be 184 times higher than that of infections caused by producers (p = 0.0012). In contrast to Model 1, *S. aureus *did not differ from *S. epidermidis *in terms of the probability of peritonitis resolution (p = 0.7014), whereas the chance of cure of infections caused by other CoNS species was estimated to be 46 times higher than that of infections caused by *S. epidermidis *(p = 0.0175). Alpha-hemolysin production was an independent predictor of resolution odds, with episodes caused by non-producers presenting an 8.2 times higher chance of resolution than those caused by producers (p = 0.0423). No significant effects on the probability of peritonitis resolution were observed for the remaining enzymes and toxins.

**Table 4:** Odds comparison of peritonitis resolution by logistic regression analysis (Model 2).

Factor	Log (Odds)	p	Odds ratio (95% CI)
Age (birth to 20 years/>60 years)	0.0602	0.97	
Age (21 to 40 years/>60 years)	4.2565	0.06	
Age (41 to 59 years/>60 years)	1.9474	0.09	
Gender (male/female)	1.2461	0.37	
Race (Caucasian/non-Caucasian)	-1.3322	0.21	
Diabetes mellitus (no/yes)	-0.6711	0.47	
System (standard/double bags)	2.3397	0.12	
Treatment time on CAPD			
<1 year	0.0531	0.98	
>1 year	-0.3045	0.77	
Treatment with vancomycin			
Oxacillin susceptible/oxacillin resistant	1.6316	0.15	
No treatment with vancomycin			
Oxacillin susceptible/oxacillin resistant	10.0189	0.004	23,906 (25.4; exp{16.9274})
Etiological agent			
*S. epidermidis S. aureus*	-1.6481	0.70	
Other CoNS/*S. epidermidis*	3.8238	0.017	46 (2.0; 1,069.4)
Slime production (no/yes)	5.2149	0.001	184 (7.8; 4,354.2)
Enzyme production (no/yes)			
α-Hemolysin	2.1092	0.04	8.2 (1.1; 63.1)
TNAse	2.0500	0.28	
Lecithinase	3.5545	0.06	
Toxin production (no/yes)			
Enterotoxin A	2.3914	0.26	
Enterotoxin B	0.5669	0.13	
Enterotoxin C	4.0501	0.06	
TSST-1	1.3694	0.48	

## Discussion

In the present study we investigated new episodes of staphylococcal peritonitis in PD patients and compared episodes caused by *S. aureus*, *S. epidermidis *and other CoNS, focusing on the role of virulence factors in peritonitis outcome. CoNS were the most frequent etiological agent, in agreement with other studies. In addition to *S. epidermidis*, seven other CoNS species were identified, the most predominant being *S. haemolyticus*. This is an important finding since studies regarding other CoNS are scarce in the literature.

The resolution rate of episodes caused by *S. aureus *was lower than that of infections caused by CoNS. Similar findings have been reported in the prospective studies of Bunke et al [5] and Peacock et al [6], and in the recent retrospective study of Davenport [1]. Since the rate of oxacillin resistance was higher among CoNS strains than among *S. aureus *strains, a contribution of drug resistance is unlikely. In fact, our results showed no difference in resolution rates between episodes caused by oxacillin-susceptible and oxacillin-resistant strains.

All patients received an intermittent regimen of cefazolin plus amikacin as initial treatment. Some investigators [28,29] argue that the continuous addition of beta-lactams to the PD fluid is more effective for the treatment of CoNS infections by overcoming moderate bacterial resistance. Since a higher resolution rate was observed for CoNS episodes compared to *S. aureus *episodes and the number of strains presenting moderate resistance to oxacillin was low, it is unlikely that the antibiotic regimen has influenced the results.

Regression analysis using the two models showed no influence of age, race, gender, CAPD treatment time, diabetes, or exchange system on the progression of peritonitis. Similar results have been reported by Krishnan et al [7], except for the influence of race and dialysis treatment time.

Slime production was independently associated with non-resolution of peritonitis. This finding agrees with data on CoNS infections previously published by our group [15] and with the results of Kristinsson et al [13]. The latter authors proposed that slime production may promote bacterial adherence to catheters, facilitating colonization and peritonitis relapse. Thus, slime production might be a virulence factor and simultaneously worsens the response to infection, protecting bacterial cells from the host's natural defense mechanisms and from the action of antibiotics.

In contrast to Model 1, no difference in the probability of peritonitis resolution was observed between *S. aureus *and *S. epidermidis *when Model 2 was used. This finding might be explained by the fact that inclusion of enzymes and toxins in the model permitted the control for the effect of species factor on these bacterial products. In other words, the species effect observed in the first model for *S. aureus *episodes was not separated from the pathogenicity factors included in the second model. In addition, whereas the chance of cure of *S. epidermidis *episodes compared to other CoNS infections tended to be lower in the first model, the second model showed a significantly lower probability of cure of *S. epidermidis *episodes, an independent outcome not influenced by toxin or enzyme production.

Among the virulence factors studied, only α-hemolysin production was significantly and independently associated with a higher probability of non-resolution. Recent data published by Haslinger-Löffler et al [30] suggest that α-hemolysin plays a specific role in the pathogenesis of peritonitis. Using cultured human peritoneal mesothelial cells, these authors showed that the *S. aureus *subgroup characterized as invasive and α-hemolysin producing induced caspase-independent cell death. Unlike *S. aureus*, no cytotoxic effects were triggered by any of the *S. epidermidis *strains which were noninvasive and did not produce α-hemolysin. These findings, together with our results, suggest that this enzyme plays a pathogenic role in PD-associated peritonitis. Since mesothelial cells participate in the early host defense against infections [31], damage caused by α-hemolysin may contribute to the poor course of *S. aureus *peritonitis.

This study has some limitations, particularly the small number of cases, which reduce its statistical power and prevented the separate analysis of *S. aureus *and CoNS peritonitis episodes. Further studies with bigger number of cases are necessary to overcome this limitation and to confirm the present results.

## Conclusions

In conclusion, host factors, as well as dialysis treatment time and exchange system, probably have little or no influence on the response to PD-associated peritonitis treatment. However, the prognosis of these infections is strongly influenced by characteristics of the causative agent such as species and virulence factors. Although these factors may act in concert, our data suggest that slime and α-hemolysin production independently contribute to poor peritonitis outcome.

## Competing interests

The authors declare that they have no competing interests.

## Authors' contributions

PB participated in the design and coordination of the study and in the collection of the clinical data, analyzed the data, and wrote and revised the manuscript. ACM participated in the design of the study and performed the E-test. JENB performed the microbiological tests. JCTC participated in the collection of the clinical data and contributed to the design of the study. MLRSC participated in the coordination of the study, supervised the laboratory work, reviewed and approved the analyses, and contributed to the writing and revision of the paper. All authors read and approved the final manuscript.

## Pre-publication history

The pre-publication history for this paper can be accessed here:

http://www.biomedcentral.com/1471-2334/9/212/prepub
